# Promising Effect of High Dose Ambroxol Treatment on Neurocognition and Motor Development in a Patient With Neuropathic Gaucher Disease 2

**DOI:** 10.3389/fneur.2022.907317

**Published:** 2022-06-06

**Authors:** Charlotte Aries, Benjamin Lohmöller, Stephan Tiede, Karolin Täuber, Guido Hartmann, Cornelia Rudolph, Nicole Muschol

**Affiliations:** ^1^Department of Pediatrics, International Center for Lysosomal Disorders, University Medical Center Hamburg-Eppendorf, Hamburg, Germany; ^2^University Children's Research, University Medical Center Hamburg-Eppendorf, Hamburg, Germany; ^3^Department of Child and Adolescent Psychiatry and Psychotherapy, University Medical Center Hamburg-Eppendorf, Hamburg, Germany; ^4^Centogene GmbH, Rostock, Germany

**Keywords:** lysosomal storage disorder, neuropathic Gaucher disease, glucocerebrosidase, high dose ambroxol, pharmacological chaperone, enzyme replacement therapy

## Abstract

Gaucher Disease (GD) 2 is a rare inherited lysosomal disorder. Early-onset and rapid progression of neurovisceral symptoms lead to fatal outcome in early childhood. Treatment is symptomatic, a curative therapy is currently not available. This prospective study describes the clinical and biochemical outcome of a GD 2 patient treated with high dose ambroxol from the age of 4 months. Due to progressive hepatosplenomegaly additional enzyme replacement therapy was required 1 year after ambroxol monotherapy was initiated. Detailed clinical follow-up data demonstrated an age-appropriate neurocognitive and motor development but no clear benefit on peripheral organs. Glucosylsphingosine (Lyso-GL1) in cerebrospinal fluid decreased remarkably compared to pre-treatment, whereas Lyso-GL1 and chitotriosidase in blood increased. Ambroxol treatment of patient fibroblasts revealed a significant increase in β-glucocerebrosidase activity *in vitro*. To our knowledge, this is the first report of a GD 2 patient with age-appropriate cognitive and motor development at 3 years of age. Combination of high dose ambroxol with ERT proved to be a successful approach to manage both visceral and neurological manifestations.

## Introduction

Gaucher disease (GD) is a rare autosomal recessive lysosomal storage disorder. It is caused by mutations in the *GBA*-gene leading to a deficiency of β-glucocerebrosidase (GCase) and consecutive accumulation of its substrate glucosylceramide (GluCer) and its deacetylated form, glucosylsphingosine (Lyso-GL1) in macrophages (1). The incidence is estimated as 1:40,000–60,000 (2).

The disease is categorized into three subtypes. GD 1 is the most frequent visceral form, presenting with hepatosplenomegaly (HSM), pancytopenia and bone lesions due to accumulation of Gaucher cells in spleen, liver and bone marrow. Rapid progressive GD 2 and chronic progressive GD 3 are characterized by additional neurodegeneration. Due to an overlap in symptomology, the subtypes are today considered as a phenotypic continuum (3, 4).

Neuropathic GD 2 accounts for ~2 % of GD cases. It is characterized by onset in early infant age (median 3–6 months), a severe and rapid progressive neurological impairment and a fatal outcome within the first years of life. Average survival is reported as 11–19 months (range 2–55 months) (5, 6).

Neurological involvement in GD 2 comprises abnormal muscle tone, consisting of either muscular hypertonia or hypotonia of varying degree, progressive cognitive decline, bulbar and pyramidal signs as well as epilepsy with myoclonic seizures (6). Ophthalmologically, strabismus, ophthalmoparesis, ocular apraxia and impaired vision have been reported (5, 7). Another classical sign for GD 2 is neonatal ichthyosiform erythroderma, also referred to as “collodion baby” phenotype (8, 9). Common findings include mild cerebral atrophy on brain MRI and abnormal brain stem auditory and visual evoked response testing (BAER and VER) (10, 11).

To date there is no approved therapy for GD 2. First line therapies for GD 1 and 3 comprise enzyme replacement therapy (ERT) and substrate reduction therapy (SRT). While both have shown significant impact on visceral manifestations, including improvement of HSM, pancytopenia and bone involvement, no positive effect on neurological symptoms has been reported (12–14).

Pharmacological chaperone therapy is an emerging new strategy for the treatment of lysosomal storage disorders. By stabilization of misfolded proteins, chaperones improve intracellular trafficking and increase enzyme activity (15). Another important characteristic is their potential to target the central nervous system (CNS) by overcoming the blood brain barrier (16, 17).

Ambroxol, a secretolytic expectorant approved for the treatment of acute and chronic bronchopulmonary diseases and hyaline membrane disease in newborns, has been identified as a pharmacological chaperone in neuropathic GD (18). The impact of ambroxol on GCase activity in healthy mice and cultured GD patient skin fibroblasts has been investigated. Stimulation with ambroxol led to a significant increase in enzyme activity in several organs of the mice, including the cerebellum. Furthermore, a mutation-dependent increase of GCase activity was demonstrated in treated GD patient's fibroblasts (19).

A first pilot study using high dose ambroxol in humans was published in 2016 (20). Five patients, four of them being considered as GD 3 and one as GD 2, were treated with high dose ambroxol in combination with ERT. The results indicated good safety and tolerability as well as a wide drug distribution into different organs including the central nervous system. Furthermore, positive effects on biochemical markers [e.g., increased GCase activity in lymphocytes and decreased Lyso-GL1 in cerebrospinal fluid (CSF)] were found. Clinically, treatment with high dose ambroxol improved generalized myoclonus, seizures and pupillary light reflex dysfunction.

This prospective study describes the clinical and biochemical outcome of a GD 2 patient treated with high dose ambroxol from the age of 4 months. Detailed developmental assessments demonstrated an age-appropriate neurocognitive and motor development within the first 34 months of life. Biochemical studies in patient lymphocytes and fibroblasts reflect the encouraging effect of this treatment regime. Combination of high dose ambroxol with ERT from the age of 15 months proved to be a successful approach to manage both visceral and neurological manifestations of the today 3 years old girl with GD 2.

To the best of our knowledge, this is the first description of an early diagnosed and oligosymptomatic GD 2 patient who has received ambroxol monotherapy at an early stage of disease demonstrating a remarkable outcome on neurocognitive and motor function.

## Methods

### Baseline Patient Characteristics

The patient was a female newborn and the first child of consanguineous Turkish parents. She was born after an unremarkable pregnancy at 38 weeks of gestation in a peripheral hospital and presented with a collodion baby phenotype (ichthyosis). Apart from the dermatological findings, postnatal adaptation was uneventful. Work-up of differential diagnoses for ichthyosis revealed a reduced GCase activity (0.1 μmol/l/h - Ref. > 3.2 μmol/l/h), indicating Gaucher disease. Genetic analysis detected the homozygous missense mutation p.R398L in the *GBA*-gene and confirmed the diagnosis of GD 2. Parents were shown to be heterozygous carriers of this mutation. Written informed consent for genetic analysis was obtained from both parents.

The first presentation in our pediatric outpatient clinic took place at the age of 3 months.

### Ambroxol Treatment

Ambroxol treatment was carried out according to the pilot study for neuropathic GD by Narita et al. (20). Ambroxol hydrochloride (Ambroxol Ratiopharm Solution, 7.5 mg/ml) was administered at a high dose of 25 mg/kg body weight per day, divided into three doses. In contrast, the approved dosage of ambroxol as a secretolytic agent for children under the age of 2 years is a maximum of 15 mg per day.

### Clinical Follow-Up

Clinical follow-up data was collected during routine visits in the pediatric outpatient clinic. Starting from the age of 3 months the visits took place weekly and were expanded to monthly visits after 10 weeks. Data on medical history, including adverse events and concomitant medication, were analyzed to assess the safety of ambroxol treatment. Physical examination, blood laboratories and abdominal ultrasounds were performed every month. Electrocardiography (ECG) was conducted every 2–3 months. Additionally, echocardiographies were carried out at baseline, weeks 24, 52, and 104 of ambroxol treatment.

For neurological assessment magnetic resonance imaging of the brain (cMRI) was performed at baseline, weeks 52 and 104 of ambroxol treatment. Auditory evoked potentials (AEP) and visual evoked potentials (VEP) were only conducted at baseline. Electroencephalography (EEG) was scheduled at baseline and weeks 16, 24, 52, and 104 after initiation of treatment.

Neurocognitive and motor development was evaluated using different standardized tests. The Denver Developmental Screening Test (Denver Test) and the Alberta Infant Motor Scale (AIMS) were carried out and video-taped every month during the first year of ambroxol treatment. The Bayley Scales of Infant and Toddler Development^®^, Third Edition (BSID-III) and Vineland Adaptive Behavior Scales^®^, Second Edition (VABS-II) were used to analyze neurocognitive development at the age of 15, 28, and 34 months.

Biweekly intravenous enzyme replacement therapy (ERT) with Imiglucerase (40 IE per kg body weight Cerezyme^®^, Sanofi-Aventis, Germany) was started at 15 months of age (11 months after initiation of high dose ambroxol treatment).

### Biochemical Follow-Up

#### Chitotriosidase and Lyso-GL1 Biomarker Analysis

Chitotriosidase and Lyso-GL1 were analyzed in monthly intervals during the first year of treatment and thereafter every 3–6 months. Samples were collected as EDTA blood and then analyzed in dried blood spots (DBS) and plasma for Lyso-GL1 and chitotriosidase, respectively. Cerebrospinal fluid (CSF) was obtained at baseline, month 6, 12, and 24 of ambroxol treatment. Lyso-GL1 was analyzed by Centogene (Rostock, Germany) and chitotriosidase by Biochemisches Labor (Villa Metabolica, Universitätsmedizin Mainz, Germany).

#### β-Glucocerebrosidase Activity in Patient Lymphocytes

β-glucocerebrosidase activity was determined in lymphocytes isolated from EDTA blood samples of the patient and a healthy control. Lymphocytes were enriched using Bicoll separating solution (Biochrome) according to the manufacturer's guidelines. The enriched lymphocyte fraction was resuspended in 200 μl of 20 mM HEPES buffer supplemented with protease inhibitor cocktail (cOmplete mini, Roche). Lymphocytes were lysed by four cycles of freezing and thawing. The protein was quantified using the standard Bradford assay. Therefore, lysates were adjusted to 20 μg protein in 40 μl ddH_2_O per sample, supplemented with 40 μl reaction buffer (20 mM 4-MU-β-glucopyranoside; 0.2 M Na-Citrat, pH 4.6; 0.2 % Triton X-100; 300 mM NaCl; 2 % BSA). The samples were incubated at 37 °C for 60 min. The reaction was stopped by adding 120 μl of 0.4 M glycine sodium hydroxide buffer (pH 10.4). Fluorescence was measured using a Clario Star Fluorescence Spectrometer (BMG labtech) at excitation wavelength of 355 nm and emission wavelength of 460 nm. Values are given as detected fluorescence units.

#### *In vitro* Ambroxol Treatment of Patient-Derived Skin Fibroblasts

Skin fibroblasts from the patient and a healthy donor were cultured in DMEM (Gibco) supplemented with 10 % FBS (PAN Biotech) at 37 °C and 5 % CO_2_. For the investigation of ambroxol effects on stabilizing β-glucocerebrosidase, skin fibroblasts were cultured for 4 days in 6-well plates in culture medium supplemented with ambroxol hydrochloride (Sigma-Aldrich) at 0, 3, 10, and 40 μM. For preparation of cell lysates, cells were washed with ice-cold, phosphate-buffered saline and harvested with 20 mM HEPES buffer supplemented with protease inhibitors. Cells were lysed by four cycles of freezing and thawing. Protein concentrations and β-glucocerebrosidase activities were measured as described above.

## Results

### Clinical and Laboratory Work-Up Prior to Ambroxol Treatment

Clinical examination of the patient at 3 months of age revealed moderate neurological abnormalities, particularly a discreet tendency to hyperextend the upper and lower limbs as well as a moderate hyperexcitability (see Supplementary Video). Muscle tone was elevated. Muscular and gripping reflexes were adequate and head control was normal. Growth and weight development was normal. The ichthyosis was treated with a topical hydrophobic ointment, yet the skin still showed a residual dryness which is in accordance with the natural course of the dermopathy in GD 2 (21).

Laboratory analyses revealed a normal full blood count and slightly elevated transaminases (AST 110 U/l, ALT 69 U/l). Metabolic testing demonstrated low GCase-activity of 52.65 pmol/spot^*^20 h (Ref. 200–2,000 pmol/spot^*^20 h) in dried blood spots and increased blood biomarkers (chitotriosidase 2913 nmol/ml/h - Ref. 20–150 nmol/ml/h; Lyso-GL1 322,0 ng/ml - Ref. < 10,0 ng/ml).

### Clinical and Laboratory Follow-Up During Ambroxol Treatment

High dose ambroxol treatment (25 mg/kg body weight per day) was started at 4 months of age and was well tolerated. Apart from increased mucus production during the first couple of weeks, the patient showed no side effects or signs of toxicity and the parents reported no adverse events. One week after initiation of high dose ambroxol treatment the patient presented with high-pitched screaming and hyperextension in prone position. These neurological signs improved during the following weekly assessments. Three months after initiation of treatment, neurological examination was normal and none of these symptoms were reported by the parents anymore.

**Figure 1:**
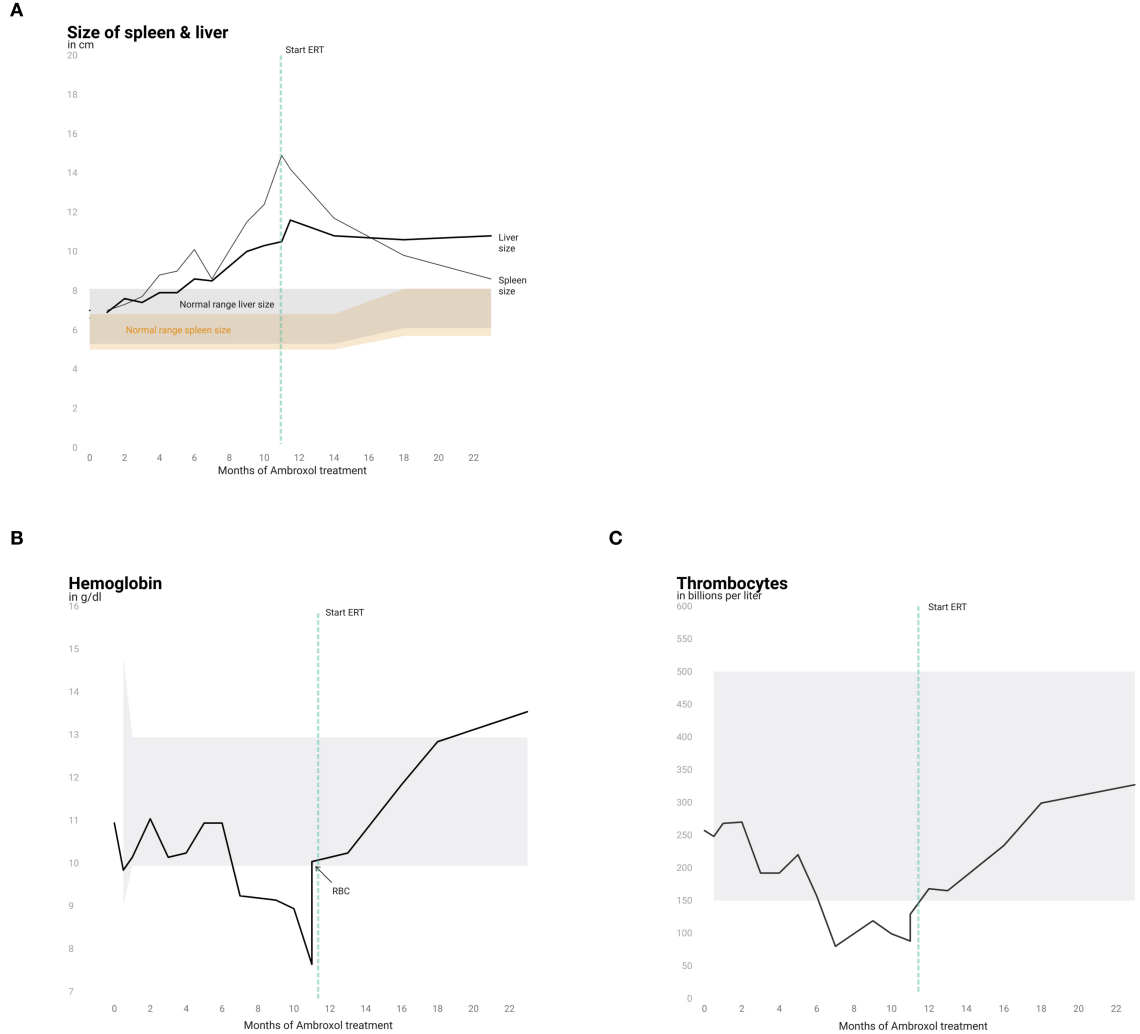
Liver and spleen size and hematological parameters. Progression of spleen and liver size **(A)** and analysis of hemoglobin **(B)** and thrombocytes **(C)** over the course of 22 months. Normal ranges are shown in gray (liver, hemoglobin and thrombocytes) and in orange for spleen size.

Monthly physical examination and abdominal ultrasound revealed increasing HSM within the first 11.5 months of ambroxol treatment (Figure 1A). Hematological parameters remained stable for up to 6 months after initiation of ambroxol treatment (Figures 1B,C) and decreased hereafter. Due to progressive anemia and thrombocytopenia at 15 months of age (11.5 months after initiation of ambroxol treatment) the patient received a red blood cell transfusion and was started on ERT with Imiglucerase. Hematological parameters quickly recovered in the following weeks. Spleen size showed a marked decline, while liver size mildly decreased and then stabilized (Figure 1A).

**Figure 2:**
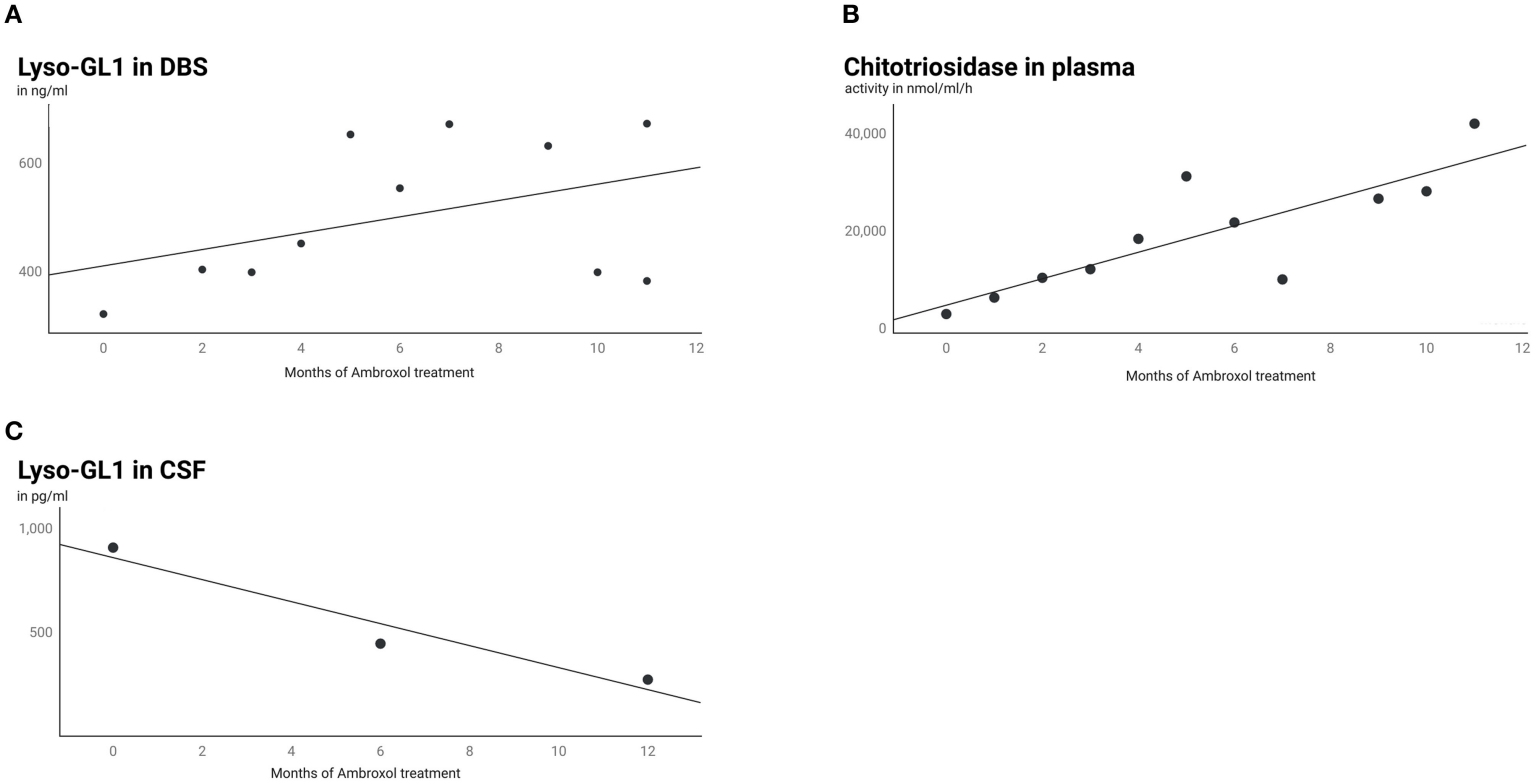
Lyso-GL1 in DBS, Chitotriosidase in plasma and Lyso-GL1 in CSF. Continuous increase of Lyso-GL1 in DBS **(A)** and Chitotriosidase in plasma **(B)** over 12 months of ambroxol monotherapy and in contrast a continuous decrease of Lyso-GL1 in CSF **(C)** over 12 months of ambroxol monotherapy and another 12 months of combination of ambroxol and ERT.

HSM was accompanied by increasing biomarkers in serum (Figures 2A,B). Chitotriosidase and Lyso-GL1 blood concentrations showed an overall increase from baseline (Lyso-GL1 322 ng/ml, Chitotriosidase 2,913 nmol/ml/h) to month 11.5 (Lyso-GL1 673 ng/ml, Chitotriosidase 42,015 nmol/ml/h) despite ambroxol monotherapy. In contrast, the concentration of Lyso-GL1 in CSF continuously decreased from baseline (908 pg/ml) to month 24 (25.8 pg/ml) (Figure 2C).

ECGs and echocardiographies displayed normal cardiac anatomy and function during 2 years of treatment. EEGs demonstrated unremarkable results as well. At baseline, the patient's cMRI was assessed as age-appropriate, while follow up at 52 and 104 weeks showed white matter hyperintensities interpreted as compatible with GD 2. Analysis of AEP at baseline was normal, whereas the analysis of VEP showed transmission delays. Taking into account the lack of standard VEP values for infants it remains difficult to assess whether this should be considered as pathologic. Ophthalmologic examination was unremarkable.

### Neurocognitive and Motor Development

Neurocognition was assessed by BSID-III and VABS-II in German language and demonstrated an age-appropriate cognitive development for up to 15 months of age (with a developmental quotient (DQ) of 100). At 28 and 34 months of age the BSID-III suggested a slight cognitive delay with a DQ of 96 and 94, respectively. In contrast, receptive and expressive language skills were below-average (Tables 1A,B). As the child is raised bilingually and Turkish is the predominant family language, this might have influenced the outcome of the testing over time. The results of the VABS-II showed a developmental delay in all categories with the exception of a nearly age-appropriate motor development. Denver tests revealed age-appropriate milestones and AIMS assessments confirmed age-appropriate progress in all domains (Table 1C). Regular video-taping displayed age-appropriated motor development and creative play up to 3 years of age (see Supplementary Video).

**Table 1:** Neurocognitive and motor development assessments.

**(A) Neurocognitive development, Bayley Scales of Infant and Toddler Development** ^**®**^ **, Third Edition (BSID-III)**			
**Domain**	**AEqs in mo at age of 15 mo**	**AEqs in mo at age of 28 mo**	**AEqs in mo at age of 34 mo**
Cognitive development	15	27	32
Receptive language	15	17	21
Expressive language	10	12	18
Fine motor	15	25	32
Gross motor	12	19	26
**(B) Adaptive behavior, Vineland Adaptive Behavior Scales** ^®^ **, Second Edition (VABS-II)**			
**Domain**	**AEqs in mo at age of 15 mo**	**AEqs in mo at age of 28 mo**	**AEqs in mo at age of 34 mo**
Communication	10,5	15	22
Daily life skills	13	20	26
Social	9	19	23
Motor	12	24,5	34
Adaptive behavior (Standard score)	11 84 (80–88)	20 86 (83–89)	26 87 (84–90)
**(C) Alberta Infant Motor Scale (AIMS) and Denver Test**			
**Mo after initiation of ambroxol**	**Age in mo**	**AIMS percentile ranks**	
0	4	5	
1	5	10	
2	6	10	
3	7	5	
4	8	10	
5	9	5	
6	10	5	
7	11	50	
8	Not done	Not done	
9	12.5	25	
10	14	25	
11	14.75	<5	
12	15.8	10	

*AEq, Age equivalent; mo, months; Standard scores (mean = 100, standard deviation = 15)*.

### GCase Enzyme Activity and Biomarker Analyses

**Figure 3:**
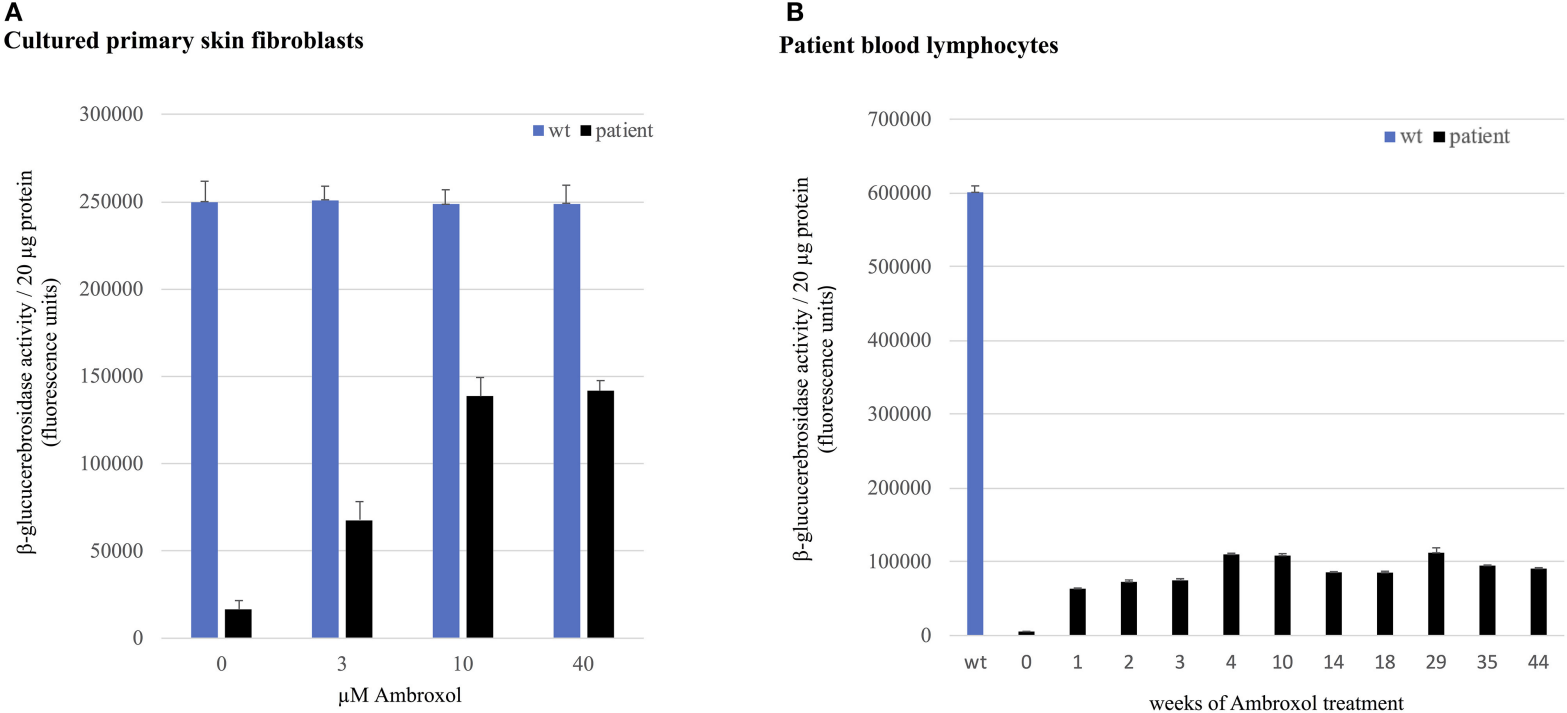
GCase activity in skin fibroblasts and lymphocytes. GCase activity in **(A)** patient's primary skin fibroblasts compared to healthy donor cells (wild-type, wt) and **(B)** in patient lymphocytes compared to healthy donor cells (wt) at weeks 0–44 of ambroxol treatment.

*In vitro* experiments in patient's fibroblasts isolated prior to ambroxol treatment demonstrated a significant dose dependent response to ambroxol. GCase activity showed an increase of up to 57 % of wild-type activity, reaching a plateau at a concentration of 10 μM ambroxol hydrochloride (Figure 3A).

GCase activity in patient lymphocytes analyzed in weeks 1–44 of ambroxol therapy showed an up to 20-fold increase compared to pre-treatment corresponding to 19 % of GCase activity in wild-type lymphocytes (Figure 3B).

## Discussion

GD 2 is a severe and rapid-progressive lysosomal disorder with early-onset of neurovisceral symptoms and a fatal outcome in early infancy (5, 6). Neonatal ichthyosis, also known as a collodion baby phenotype, might occur as a first symptom of GD 2 even before neurological manifestations become apparent (22, 23). The patient described here presented as a collodion baby at birth and developed moderate neurological symptoms, including hyperexcitability and hyperextension of the upper and lower limbs, by 3 months of age. Genetic analysis revealed the homozygous mutation p.R398L (c.1193G>T) in the *GBA* gene. Bulut et al. previously reported on a GD 2 patient with the identical homozygous mutation who demonstrated ichthyosis, severe neurological impairment (irritability and hypotonia), massive HSM and failure to thrive at 9 months of age. Treatment was symptomatic, consisting of multiple transfusions and splenectomy. The patient died at the age of 18 months due to an aspiration pneumonia (24).

To date, no curative treatment is available for GD 2. Therapeutic options for GD 1 and 3 comprise ERT and SRT. While ERT successfully addresses visceral and hematological symptoms, the enzyme is not able to cross the BBB, hence no clear benefit regarding CNS involvement is to be expected (11, 25). Miglustat, an approved SRT in GD, is known for overcoming the BBB. Nonetheless, it has not shown any significant positive effect on neurological symptoms in GD 3 (13). Eliglustat, another drug for SRT, is approved for treatment of GD in adult patients only. Supportive treatment in GD 2 may address feeding difficulties, failure to thrive, gastroesophageal reflux, muscle spasms or abnormal movements, choking and irritability.

The pharmacological chaperone ambroxol seems to be a promising candidate for treating neuropathic GD. Ambroxol is a small molecule that binds in a mutation-dependent manner to misfolded proteins in the ER and facilitates the shuttle to the lysosome. Furthermore, by crossing the BBB it might have an impact on central nervous system manifestations (20). Due to the encouraging results of the above mentioned pilot study, ambroxol has become a common off label therapy for neuropathic GD (26). In a recently published analysis of 23 GD 2 patients receiving different approved and experimental treatment regimen (symptomatic, ERT, SRT, HSCT, N-acetylcysteine and ambroxol) the mean age of death was 19.2 months (3–55 month) (6). Although some of these patients got diagnosed and started on treatment very early, all of them displayed respiratory involvement, failure to thrive, abnormal muscle tone and developmental impairment. Two of these patients received a monotherapy with ambroxol and died at the age of 3 and 17 months, respectively. Three patients were treated with a combination of ERT and ambroxol. In January 2020 they were still alive and aged 17, 27, and 74 months, suggesting a positive effect of the combination of ERT and ambroxol on the overall survival of these patients.

The patient described here was initially started on high dose ambroxol monotherapy at 4 months of age. The drug was well tolerated without any side effects or signs of toxicity. After 1 year of treatment the patient displayed an astonishing age-appropriate neurocognitive and motor development. Lyso-GL1 biomarker in CSF decreased by 97 % compared to pre-treatment. Nevertheless, Lyso-GL1 biomarker in DBS and chitotriosidase concentrations in plasma increased. Due to progressive HSM and anemia a red blood cell transfusion was indicated at the age of 15 months and the patient was additionally started on ERT. Thus, our data demonstrate an outstanding effect of ambroxol on CNS manifestations while a clear benefit on peripheral organs was missing. In accordance with the data reported by Roshan Lal et al. high-dose ambroxol treatment in combination with ERT seems to have the best benefit in GD 2 patients with specific *GBA* gene mutations. Maegawa et al. identified certain protein sequences of GCase which are susceptible to stabilization by ambroxol. Nevertheless, comprehensive clinical data on the mutation-specific outcome on ambroxol treatment is needed.

*In vitro* experiments revealed a remarkable increase of enzyme activity of 57 % and 19 % of wild-type activity in patient's fibroblasts and lymphocytes, respectively. This confirms responsiveness of the mutation p.R398L (c.1193G>T) to ambroxol. Tissue specific properties might explain the discrepancies between experimental data and clinical outcome and remain to be elucidated. Furthermore, due to a link of GCase activity and alpha-Synuclein accumulation (27), analysis of this neurodegenerative marker might provide important insights into the response of the central nervous system to ambroxol. All these findings are in accordance with a recent paper by Pantoom et al. who suggest that ambroxol does not solely act as a pharmacological chaperone but also has additional modes of action (28).

In conclusion, to the best of our knowledge, this is the first report of a GD 2 patient with a nearly age-appropriate neurocognitive and motor development after treatment with high-dose ambroxol was initiated at an early stage of disease. The meanwhile 3-year-old girl continues an unexpected development with a remarkable overall quality of life. Therefore, pharmacological chaperone therapy with ambroxol should be considered as a very promising mutation-dependent treatment option for GD 2 patients but further studies are required.

## Data Availability Statement

The original contributions presented in the study are included in the article/Supplementary Material, further inquiries can be directed to the corresponding author/s.

## Ethics Statement

Ethical review and approval was not required for the study on human participants in accordance with the local legislation and institutional requirements. Written informed consent to participate in this study was provided by the participants' legal guardian/next of kin. Written informed consent was obtained from the individual(s), and minor(s)' legal guardian/next of kin, for the publication of any potentially identifiable images or data included in this article.

## Author Contributions

CA, BL, and NM examined the patient, collected follow-up data, conceptualized the idea, and prepared the manuscript. CR and NM advised data analyses and interpretation and were involved in manuscript preparation and finalization. ST performed biochemical enzyme activity analyses. KT carried out neurocognitive and motor deveolopment assessments. GH performed Lyso-GL1 analysis. All authors read and approved the manuscript.

## Conflict of Interest

GH was employed by the company Centogene GmbH. The remaining authors declare that the research was conducted in the absence of any commercial or financial relationships that could be construed as a potential conflict of interest.

## Publisher's Note

All claims expressed in this article are solely those of the authors and do not necessarily represent those of their affiliated organizations, or those of the publisher, the editors and the reviewers. Any product that may be evaluated in this article, or claim that may be made by its manufacturer, is not guaranteed or endorsed by the publisher.
